# Comparison of Commercial Lateral Flow Immunochromatography with Phenotypic and Genotypic Assays for the Detection of Carbapenemase-Producing Gram-Negative Bacteria at Tanta University Hospitals

**DOI:** 10.3390/microorganisms14010031

**Published:** 2025-12-22

**Authors:** Marwa S. Taha, Basant Mostafa Gabr, Wafaa Abd Elaziz, Ahmed Mostafa Elgohary, Bsant S. Kasem, Reham M. Elkolaly, Hytham I. S. Elatrozy, Marwa N. Emam, Asmaa S. Essawy, Heba E. M. Sharaf Eldin, Rehab A. Mohamed, Mahmoud Z. Elkadeem, Sherif Abdelbaky, Mona Abd El-Aziz Gadallah

**Affiliations:** 1 Department of Medical Microbiology and Immunology, Faculty of Medicine, Tanta University, El-Geish St., Medical Campus, Tanta 31527, Egypt; basant.mostafa@med.tanta.edu.eg (B.M.G.); mona.gadallah@med.tanta.edu.eg (M.A.E.-A.G.); 2 Department of Biochemistry, Genetic Engineering and Biotechnology Division, National Research Center, Dokki, Giza 12622, Egypt; fofa991@yahoo.com; 3 Department of Clinical Pathology, Faculty of Medicine, Tanta University, Tanta 31527, Egypt; ahmed.elgohary@med.tanta.edu.eg (A.M.E.); bsantsafwat16120167@gmail.com (B.S.K.); 4 Department of Chest, Faculty of Medicine, Tanta University, Tanta 31527, Egypt; r.elkolaly@med.tanta.edu.eg; 5 Department of Neurosurgery, Faculty of Medicine, Tanta University, Tanta 31527, Egypt; haytham_atrozy@med.tanta.edu.eg; 6 Department of Physiology, Faculty of Medicine, Tanta University, Tanta 31527, Egypt; marwa.emam@med.tanta.edu.eg; 7 Department of Biophysiology, Ibn Sina National College for Medical Studies, Jeddah 22421, Saudi Arabia; 8 Department of Anatomy, Faculty of Medicine, Tanta University, Tanta 31527, Egypt; asmaa-saeed@ibnsina.edu.sa; 9 Department of Anatomy, Ibn Sina National College for Medical Studies, Jeddah 22421, Saudi Arabia; 10 Department of Basic Dental and Medical Sciences, Faculty of Dentistry, Mutah University, Al-Karak 61710, Jordan; hebasharaf@mutah.edu.jo; 11 Department of Histology and Cell Biology, Faculty of Medicine, Tanta University, Tanta 31527, Egypt; 12 Department of Family Medicine, Faculty of Medicine, Suez Canal University, Ismailia 41522, Egypt; rehabali79@ibnsina.edu.sa; 13 Department of Family and Community Medicine Department, Ibn Sina National College for Medical Studies, Jeddah 22421, Saudi Arabia; 14 Department of Tropical Medicine and Infectious Disease, Faculty of Medicine, Tanta University, Tanta 31527, Egypt; mahmoud.elkadeem@med.tanta.edu.eg (M.Z.E.); smabdelbaky@moh.gov.sa (S.A.); 15 King Fahd Specialist Hospital, Buradyah 52366, Saudi Arabia

**Keywords:** carbapenemase-producing *Enterobacterales* (CRE), carbapenemase genes, lateral flow immunoassays (LFIAs), *bla*_NDM_, *bla*_OXA_, *bla*_IMP_, *bla*_KPC_, *bla*_VIM_

## Abstract

It is crucial to identify Enterobacterales that produce carbapenemase to treat and manage hospital infections. The suggested techniques for their identification need a lengthy wait, technical knowledge, and training. Lateral flow immunoassays (LFIAs) provide a solution to these requirements. Thus, this study compared LFIA with phenotypic and genotypic tests for carbapenemase-producing bacteria. Fifty clinical isolates of carbapenem-resistant superbugs were examined. KPC, VIM, NDM, IMP, and OXA-48-like enzymes were evaluated and compared with phenotypic tests and LFIA. Regarding the phenotypic characteristics, the mCIM was positive in 37/50 (74%), and the eCIM was positive in 21/50 (42%). Regarding using LFIA, 41 out of the total isolates (82%) gave a positive red line with one or more of the tested genes. The most frequently detected gene was *bla*_NDM_ (27/50 (54%)), and the least detected one was *bla*_IMP_ (14/50 (28%)), which was in accordance with the PCR results. While investigating the accuracy of LFIA vs. PCR, it was found that LFIA had 100% sensitivity in the detection of the *bla*_NDM_ and *bla*_OXA_ genes, with 85.2% and 91.4% specificity, respectively, while for the *bla*_IMP_, *bla*_KPC_, and *bla*_VIM_ genes, the values were 91.7% and 92.1%, 94.1% and 90.9%, and 95.5% and 89.3%, respectively. The overall accuracy of LFIA ranged from 92 to 94%. Our comparison with molecular assays revealed remarkable agreement, so we propose that this test might be utilized as a supplementary tool.

## 1. Introduction

The carbapenem class of antibiotics is broadly utilized in hospitals for treatment of critical infections triggered by multidrug-resistant (MDR) pathogens. Nowadays, the boosted consumption of carbapenems has led to the emergence and broad dissemination of carbapenem resistance, particularly in high-risk places, such as intensive care units [1].

Infections by carbapenemase-producing *Enterobacterales* (CRE) and other Gram-negative superbugs pose a significant worldwide health hazard, owing to their high treatment costs, high mortality, and restricted remaining effective therapeutic agents, with a subsequent threat to revert humanity to a pre-antibiotic era [2].

Resistance to carbapenems is chiefly due to production of carbapenemase enzymes that are encoded by highly transferable plasmids. As stated by the Ambler Classification, β-lactamases are divided into four classes from A to D. Three classes of them have carbapenemase activity: class A, including *Klebsiella pneumoniae* carbapenemases (KPCs); class B metallo-β-lactamases (MβLs), including New Delhi metallo-beta-lactamase (NDM), imipenemase (IMP), and Verona integron-encoded metallo-β-lactamase (VIM); and class D, with oxacillinase (OXA) carbapenemases [3].

Distinguishing carbapenemase production is crucial in clinical microbiology laboratories for a multitude of reasons, such as choosing the appropriate therapy, establishing surveillance protocols within the hospital setting, studying the epidemiology of CRE, and implementing infection control measures to restrain their spread [4].

The molecular approach represents the most successful technique for detection of carbapenemase genes. Nevertheless, it frequently necessitates specialized, costly laboratory infrastructure, rendering it unattainable in several resource-constrained environments and smaller medical facilities. Moreover, this technology detects the existence of resistance genes but does not provide results of susceptibility testing or the level of resistance gene expression. Additionally, genetic variability in carbapenemase genes may restrict molecular identification of CRE, leading to phenotypic resistance unrevealed by gene sequence assays [5].

There is a necessity to find a quick, accurate, point-of-care, and low-priced alternative phenotypic test for the diagnosis of CRE [6]. Lateral flow immunoassay assay (LFIA) is an immunochromatographic method that uses antibodies to directly identify specific carbapenemase enzymes. It has been established with the aim of having a prompt and straightforward approach for recognizing carbapenemases [7]. Moreover, LFIAs are simple techniques that require limited technical expertise in the absence of expensive equipment and are generally less expensive than genotypic methods [8].

Consequently, the aim of this study was to evaluate the performance of LFIA in comparison with phenotypic and genotypic assays for the detection of carbapenemase-producing Gram-negative bacteria at Tanta University Hospitals.

## 2. Materials and Methods

### 2.1. Study Design, Location, and Duration

This was a cross-sectional study that was carried out in the Medical Microbiology and Immunology Department, the Faculty of Medicine, Tanta University, and the National Research Center in Egypt during the period of this research from January 2024 to December 2024.

### 2.2. Sample Size Justification

The sample size was calculated using Epi Info™ 7 to 274. The sample size was estimated based on a 20% proportion of carbapenem resistance, according to a study in Egypt [9], at a 95% level of confidence and a 5% margin of error. Assuming a 10% drop-out, the sample size was rounded to 50 cases.

### 2.3. Inclusion and Exclusion Criteria

This study was conducted on 50 carbapenem-resistant Gram-negative superbugs that were isolated from 50 non-duplicate clinical specimens from Tanta University Hospitals. Any Gram-positive or carbapenem-sensitive Gram-negative isolates were excluded.

### 2.4. Ethical Consideration

This study was approved by Tanta University’s Institutional Review Board for the Faculty of Medicine in Egypt (approval code: 36264PR601/3/24).

### 2.5. Samples Collection and Processing

All samples were gathered under complete aseptic precautions. They were adequately labeled and delivered as soon as possible to the Medical Microbiology and Immunology Department laboratory. All samples were cultured on MacConkey, nutrient agar, and blood agar plates (HiMedia, Mumbai, India). After that, Gram-stained smears were prepared and examined microscopically. All plates were incubated at 37 °C for 24 h, and then the isolates in the primary plates were identified. All samples were fully identified with further antibiotic sensitivity testing by the automated Render MS 100 culture and sensitivity system (Render Biotech Co., Ltd. (Shenzhen, China)).

### 2.6. Phenotypic Detection of Carbapenem-Resistant Gram-Negative Bacteria

Screening for carbapenem resistance was carried out using imipenem (IPM) 10 μg, ertapenem (ETP) 10 μg, and meropenem (MEM) 10 μg discs (Bioanalyse, Ankara, Turkey) by the modified Kirby–Bauer disc diffusion method on Mueller–Hinton agar (MHA) plates (HiMedia, India), according to the Clinical and Laboratory Standard Institute (CLSI) guidelines [10].

### 2.7. Detection of Carbapenemase Enzyme Production

The nominated carbapenem-resistant Gram-negative isolates were tested for carbapenemase enzyme production by phenotypic, immunochromatographic, and genotypic assays.

- Phenotypic Assay

This assay was performed by two recommended CLSI tests: the modified carbapenem inactivation method (mCIM) and the EDTA-modified carbapenem inactivation method (eCIM) [10].

II. Immunochromatographic assay (Lateral Flow Immunoassay)

The lateral flow immunoassay (Goldstream, Beijing Gold Mountain River Tech Development Co., Ltd., Beijing, China) rests on the interaction between carbapenemases and labeled anti-carbapenemase monoclonal antibodies. The assay was conducted in accordance with the manufacturer’s guidelines. The examination was conducted utilizing fresh colonies. One colony was suspended in five drops (150 μL) of extraction buffer. The bacterial culture was homogenized using a vortex, and 100 μL was applied to a nitrocellulose membrane. The suspension traversed the membrane due to capillary action and engaged with the immobilized complementary anti-carbapenemase monoclonal antibodies. Each test pack included two test cassettes, one of which contained labeled monoclonal antibodies against IMP, KPC, and NDM, while the second one contained labeled monoclonal antibodies against VIM and OXA-48, with the same abbreviations of the enzymes on the test cassette for all enzymes, except the abbreviation of OXA-48, which was O 48 on the cassette. Additionally, letter C was present on each cassette, in accordance with an internal positive control. The type of carbapenemase was defined when the red line appeared in the line with the respective abbreviations. The confirmed reaction occurred when a red line manifested at location C after a 15 min incubation period at room temperature. The data were interpreted without knowledge of the PCR outcomes to guarantee impartiality.

III. Genotypic Assay

DNA extraction was carried out by the boiling method [11]. Briefly, different strains were grown overnight at 35 °C on MacConkey agar. Two or three colonies of each culture were harvested from the surface of the agar plates and resuspended in 200 μL of sterile distilled water. The cell suspension was heated for 15 min at 100 °C and then centrifuged at 10,000 g for 10 min, and the supernatant was used as a source of template DNA for amplification of different genes by polymerase chain reaction (PCR).

Each PCR reaction had a total volume of 20 μL, comprising 2 µL of extracted DNA, 10 µL of 2X PCR Master Mix (Thermo Scientific, Lithuania, Waltham, MA, USA), 6 µL of nuclease-free water, and 1 µL of each forward and reverse primer (10 µM). The PCR protocol was executed as follows: ten minutes at 94 °C, followed by thirty cycles of amplification, each including thirty seconds at 94 °C, forty seconds at 52 °C, and fifty seconds at 72 °C, concluding with five minutes at 72 °C for the final extension. The primers used in this study are summarized in the Supplementary Data, Table S1 [12].

The PCR results were detected using electrophoresis on 2% agarose gels stained with ethidium bromide. The PCR product size was assessed under UV light utilizing a 100 bp plus DNA ladder(QIAGEN^®^, GelPilot^®^, SYBR^®^, (Molecular Probes, Inc., Dreieich, Germany)). Discrepancies between the LFA results and the PCR data were inspected.

### 2.8. Statistical Analysis

The data were fed to the computer and analyzed using the IBM SPSS software package, version 20.0. (Armonk, NY, USA: IBM Corp). Qualitative data were described using numbers and percentages. The significance of the results was judged at the 5% level.

## 3. Results

### 3.1. Distribution of Isolates

In the current study, a total of 50 carbapenem-resistant superbugs were isolated from various clinical specimens. The isolated bacteria contained two main groups: carbapenem-resistant enterobacterales (CRE) and non-fermenting Gram-negative bacteria (NFGNB). Each group included 50% (n = 25) of the total isolates. *Klebsiella pneumoniae* was the most common type of CRE isolate, making up 30% (n = 15) of the whole samples, while *Escherichia coli* made up 10% (n = 5), *Enterobacter cloacae* 6% (n = 3), and *Klebsiella oxytoca* 4% (n = 2). The NFGNB group was mostly made up of *Acinetobacter baumannii* (28% (n = 14)) and *Pseudomonas aeruginosa* (22% (n = 11)).

### 3.2. Phenotypic Detection of Carbapenemase Enzyme Production

Regarding the phenotypic detection of carbapenemase production by the mCIM and eCIM tests, the mCIM was positive in 37/50 (74%), and the eCIM was positive in 21/50 (42%), as shown in Table 1.

### 3.3. Lateral Flow Immunoassay and Genotypic Assay in Detection of Carbapenemase Enzyme Production

Regarding using LFIA and PCR for the detection of five major carbapenemase-encoding genes, 41 out of the total 50 isolates (82%) gave positive results, with one or more of the tested genes by both assays. Nine isolates (18%) yielded none of the five genes; six of them were non-fermenter bacteria, including two *Acinetobacter baumannii* and four *Pseudomonas aeruginosa* isolates. The most frequently detected gene was *bla*_NDM_ (54% by LFIA and 46% by PCR), followed by *bla*_VIM_ (48% by LFIA and 44% by PCR), while the least detected gene was *bla*_IMP_ (28% by LFIA and 24% by PCR) (Table 2).

### 3.4. Detection of Single or Multiple Carbapenemase-Encoding Genes by Lateral Flow Assay

**Figure 1:**
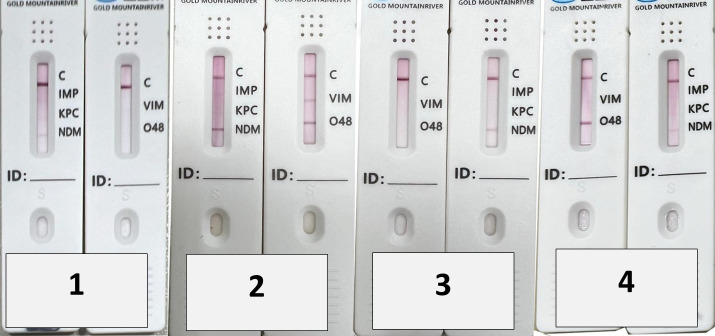
Lateral flow immunoassay for four different carbapenem-resistant isolates. Bacterial isolate no. 1 is positive for NDM; bacterial isolate no. 2 is positive for OXA-48, NDM, and VIM; bacterial isolate no. 3 is positive for NDM; and bacterial isolate no. 4 is positive for NDM and OXA-48.

Regarding the performance of the lateral flow assay in detection of single or multiple carbapenemase-encoding genes among carbapenem-resistant isolates, eleven isolates, representing 22%, contained a single gene. Double carbapenemases were identified in 12 isolates, representing 24% of the total. Triple genes were identified in eight cases (16%), whereas four genes were observed in seven (14%) isolates. Interestingly, three isolates (6%), two *Acinetobacter baumannii* isolates, and one *Klebsiella oxytoca* isolate contained all five genes (Table 3 and Figure 1).

### 3.5. Detection of Single or Multiple Carbapenemase-Encoding Genes by PCR

**Figure 2:**
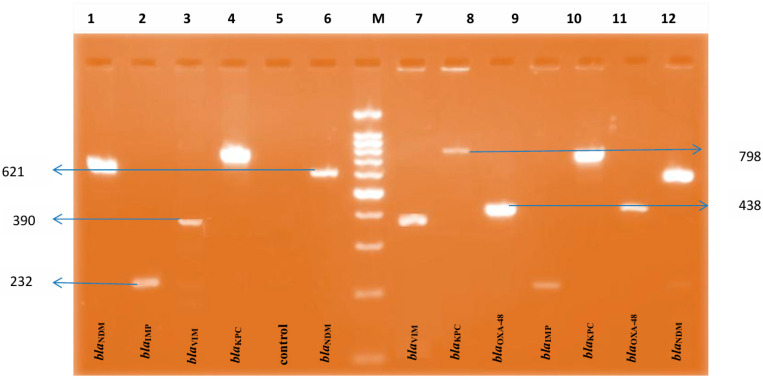
Agarose gel electrophoresis (3%) used for detection of different PCR products. Lane M: 100 bp plus DNA ladder; lanes 1,6, and 13 indicate samples with *bla*_NDM_ gene (621 bp); lanes 2 and 10 indicate samples with *bla*_IMP_ gene (232 bp); lanes 3 and 7 indicate samples with *bla*_VIM_ gene (390 bp); lanes 4 and 11 indicate samples with *bla*_KPC_ gene (798 bp); lanes 9 and 12 indicates samples with *bla*_OXA-48_ gene (438 bp); and lane 5 indicates a control sample.

Regarding the performance of PCR in detection of single or multiple carbapenemase-encoding genes among carbapenem-resistant isolates, 17 isolates, representing 34%, contained a single gene. Double carbapenemases were identified in nine isolates, representing 18% of the total. Triple genes were identified in eight isolates (16%), whereas four genes were observed in five isolates (10%). Surprisingly, two *Acinetobacter baumannii* isolates harbored all five genes (Table 4 and Figure 2).

### 3.6. Lateral Flow Assay in Comparison with PCR

While investigating the accuracy of LFIA vs. PCR, it was found that LFA had 100% sensitivity in the detection of the *bla*_NDM_ and *bla*_OXA_ genes, with 85.2% and 91.4% specificity, respectively, while the sensitivity and specificity of LFA in detection of the *bla*_IMP_, *bla*_KPC_, and *bla*_VIM_ genes were 91.7% and 92.1%, 94.1% and 90.9%, and 95.5% and 89.3%, respectively. Moreover, the overall accuracy of LFIA was 92% for all carbapenemase-encoding genes, except *bla*_OXA-48_, for which it was 94%, as shown in Table 5.

### 3.7. Phenotypic Methods in Comparison with PCR

Correlating the results of the mCIM and genotypic test, the mCIM tested positive in 35/41 (85.4%) of the PCR-positive isolates harboring one or more of the tested genes. Interestingly, only 2 out of 50 isolates were positive with the mCIM but did not harbor any of the tested genes by PCR. The sensitivity and specificity values of the mCIM in this study were 94.6% and 53.8%, respectively, while eCIM detected 21/41 (51.2%) of the PCR-positive isolates harboring one or more of the tested genes, with a sensitivity of 100% and a specificity of 31%. Especially, the eCIM detected 21/23 (91.3%) of the PCR-positive NDM isolates. The overall accuracy of the mCIM was 84%, while that of the eCIM was 60% (Table 6).

## 4. Discussion

The extreme rise in antimicrobial resistance, coupled with the shortage of current antibiotics and a lack of new antimicrobials in development, poses an urgent and ongoing threat to public health in current society. Consequently, quick and precise identification of resistant bacteria, specifically carbapenemase-producing organisms, in routine diagnostic laboratories is crucial not only for epidemiological and infection control purposes but also for the appropriate selection of antibiotic therapy, as understanding the enzyme class produced by an organism could guide therapeutic decisions [13].

Numerous immunochromatographic test kits have been created to identify the five primary carbapenemase families: KPC, NDM, IMP, VIM, and OXA-48-like [14]. Thus, this study aimed to analyze the performance of one lateral flow immunochromatography test in comparison with phenotypic and genotypic assays for the detection of carbapenemase-producing Gram-negative bacteria at Tanta University Hospitals.

In the current study, 50% of the identified carbapenem-resistant superbugs (n = 50) were CRE, and the other 50% were NFGNB. *Klebsiella pneumoniae* was the most frequent species (30%), followed by *Acinetobacter baumannii* (28%). Similarly, Mendez-Sotelo et al. [15] and Wang et al. [16] reported that *Klebsiella pneumoniae* was the most common isolate among carbapenem-resistant organisms (29.7% (25/84) and 51.8% (57/110), respectively). Additionally, Bahrami et al. [17] reported that about 50% of hospital infections were caused by *Acinetobacter baumannii* and 40% by *Klebsiella pneumoniae*. Of these, 64% and 58% of the isolates were carbapenemase-positive, respectively. Consistent with our findings, Chinese research by Zhang et al. [18] found that out of 664 carbapenem-resistant patients, 73.9% were caused by *Klebsiella pneumoniae* and 16.6% by *E. coli*. Meanwhile, the tested pathogens did not include *Acinetobacter baumannii*.

The present study highlighted the ability of the mCIM and eCIM tests to detect carbapenemase production in 74% (37/50) and 42% (21/50) of carbapenem-resistant isolates, respectively. These results are presumed as the eCIM is validated for use on Enterobacterales, but with limited extent on *Pseudomonas aeruginosa* and *Acinetobacter baumannii*, owing to significant variations in the results [10]. For illustration, the typical eCIM methodology may yield false-negative results as it inadequately identifies the MBL, and this may be due to the insufficient standard concentration of EDTA to completely block the enzyme in Pseudomonas aeruginosa [19]. Moreover, OXA-48 enzymes found in certain carbapenem-resistant *Acinetobacter baumannii* exhibit lower hydrolytic efficiency toward carbapenems compared with numerous class A and B carbapenemases; however, their presence, in conjunction with additional resistance mechanisms, like porin mutations or efflux, can result in significant carbapenem resistance [20]. Our results are analogous to those of an Indian study of 160 carbapenem-resistant isolates, in which 60.6% (n = 97) were resistant owing to carbapenemase production, as shown by mCIM positivity, while 85 isolates (87.6%) were positive for the eCIM [21]. In research by V.K. Sreeja et al. [22], 207 (94.0%) of 220 isolates tested positive for carbapenemase production, with 189 (91.2%) producing MBL. These findings are equivalent to ours. Diwakar et al. similarly found a comparable prevalence to our research [23]. However, the tiny discrepancy might be attributed to changes in the geographical location [24].

Although the gold-standard techniques for identifying carbapenemase genes are molecular techniques, the multiplex LFIA used in this study showed good matching with the PCR results. For instance, both assays detected at least one carbapenem-resistant gene in 82% of total carbapenem-resistant isolates, while 18% were negative, which were most probably resistant by other mechanisms rather than carbapenemase production.

This study noticed that the most frequently detected gene was *bla*_NDM_ (54% by LFIA and 46% by PCR), followed by *bla*_VIM_ (48% by LFIA and 44% by PCR). Meanwhile, the least detected gene was *bla*_IMP_ (28% by LFIA and 28% by PCR). Similarly, a study from South Africa found that *bla*_NDM_ was the most commonly detected carbapenemase gene among CRE (57% (469/812)), while *bla*_IMP_ was positive in only 2% (18/812) of isolates [25]. Moreover, other studies agreed with our finding of the high prevalence of *bla*_NDM_ among Gram-negative isolates [15,16]. This marked increase in *bla*_NDM_ may indicate a potential rise in these genotypes that may become endemic.

On the contrary, another study reported that *bla*_VIM_ was the highest detected gene (22.2%) among carbapenem-resistant Gram-negative isolates, followed by *bla*_IMP_ (9.7%) and then *bla*_NDM_ (4.2%) [26]. The discrepancy may result from the variance in geographically circulating strains.

The identification of infections that produce multiple carbapenemase enzymes poses a diagnostic and therapeutic challenge. For illustration, ceftazidime–avibactam is the recommended therapeutic choice for OXA-48-like-producing bacteria; however, the combination of ceftazidime–avibactam and aztreonam is favored for NDM and other MBL-producing ones. Moreover, meropenem–vaborbactam and imipenem–cilastatin–relebactam have activity solely against KPC-producing Enterobacterales.

While investigating the two, three, four, or five co-occurrences of carbapenem-resistant encoding genes by both PCR and multiplex LFIA across various CRE and NFGNB, it was found that (17/50) 34% and (11/50) 22% of the whole isolates harbored only one gene, respectively. Double carbapenemases were detected in nine (18%), triple genes were found in eight (16%), while four genes were detected in five (10%). Surprisingly, only two *Acinetobacter baumannii* isolates harbored all five genes by both assays.

These results align with another study that demonstrated that nine samples contained two or three carbapenemase genes [16]. Furthermore, MacDonald et al. found that *bla*_NDM_ and *bla*_OXA-48_ were expressed only by three isolates [13], and Rahmani et al. detected the same co-occurrence only in five isolates, while *bla*_NDM_ and *bla*_KPC_ co-occurrence was in only one *Klebsiella pneumonaie* isolate [27].

Regarding the accuracy of LFIA vs. PCR, it was found that LFIA had 100% sensitivity in the detection of the *bla*_NDM_ and *bla*_OXA-48_ genes, with 85.2% and 91.4% specificity, respectively. This indicates that the LFIA consistently detects these two genes, so if the LFIA result is negative, you can be completely certain that the bacteria lack the gene. Meanwhile, the sensitivity and specificity of LFIA in detection of the *bla*_IMP_, *bla*_KPC_, and *bla*_VIM_ genes were 91.7% and 92.1%, 94.1% and 90.9%, and 95.5% and 89.3%, respectively. Consistent with our findings, Wang et al. [16] found that a total of 93.0% of KPC (40/43), 96.3% of NDM (52/54), 100% of IMP (5/5), 100% of VIM (1/1), and 85.7% of OXA-48 (6/7) were detected with no false positives. Also, prior research assessing comparable LFA detection (four carbapenemases), OXA-48-like, KPC, VIM, and NDM revealed sensitivities ranging from 84.1% to 100% and specificities ranging from 98% to 100% [28,29,30,31,32]. Other assays for the five main carbapenemases were shown to have a sensitivity of 82.6–98.4% and a specificity of 98–100% [30,31,32]. In addition, another study evaluating the LFA against strains characterized by whole-genome sequencing discovered that the assay’s overall sensitivity was 98.07% [15]. Meanwhile, Nishida et al. [33] noticed 100% sensitivity and specificity demonstrated by the LFIAs. Furthermore, MacDonald et al. [13] stated that the assay had a global accuracy of 99.5%.

This study found a high Kappa agreement coefficient (0.793–0.865) between PCR and LFIA, indicating the assay’s usefulness in real-life applications. All kappa values were statistically significant (*p* < 0.001), confirming that the observed agreement was not due to chance. The highest level of agreement was observed for *bla*_OXA-48_ (κ = 0.865), followed by *bla*_NDM_ (κ = 0.841) and *bla*_VIM_ (κ = 0.839), while *bla*_IMP_ still demonstrated strong agreement (κ = 0.793). These findings indicate that the lateral flow assay provides highly reliable detection of carbapenemase genes when compared with PCR. This is consistent with the findings of Mendez-Sotelo et al. [15], who showed that the NG-Test CARBA 5^®^ had a high precision (kappa: 0.9) for carbapenem-resistant Enterobacterales (CRE). Additionally, Rakonjac, B., and his coworkers [34] published that the NG-Test CARBA 5 and PCR findings showed strong agreement (κ = 0.947, κ = 0.957, and κ = 0.978) in detecting NDM, OXA-48-like, and KPC, with significant *p*-values (<0.001). Furthermore, Liu P. et al. [35] stated that the overall agreement rate for the detection of five carbapenems was 99.59% (244/245). In the kappa consistency test, KPC had a kappa value of 0.990 (*p* < 0.001), while NDM, IMP, and OXA-48 all had kappa values of 1 (*p* < 0.001). Therefore, they claimed that the PCR and CARBA 5 detection methods are highly accurate and consistent in identifying various carbapenemase gene types, whether they are single- or multi-gene.

LFIA demonstrates excellent sensitivity and a high negative predictive value (NPV) for all genes, positioning it as a powerful initial screening tool (role-out tool). However, its positive predictive value (PPV) remains moderate, ranging from 78% to 87%. This PPV range signifies the presence of false-positive results. The clinical and epidemiological utility of the assay is critically dependent on whether positive outcomes can be reliably interpreted without subsequent molecular confirmation, so it is excellent for quickly confirming the absence of a carbapenemase gene, enabling timely patient management decisions (e.g., discontinuation of isolation).

Regarding the mCIM and eCIM, the sensitivity and specificity values of the mCIM in this study were 94.6% and 53.8%, respectively. In concurrence with our findings, a Mexican investigation revealed that, when compared with polymerase chain reaction (PCR), the mCIM had a 100% success rate for sensitivity, specificity, NPV, and PPV in Enterobacterales [15]. In disparity, another study reported that the sensitivity of the mCIM/eCIM to detect MBL was 89.3%, and the specificity was 98.7%, compared with genotypic PCR detection [36], in conflict with our results. Also, another study contradicted our findings by showing that the mCIM had the lowest sensitivity, at only 8.24%. An mCIM’s specificity was 40.00%, and an adjusted mCIM’s accuracy was 58.89% [37].

A limitation of this study was that we could not detect all variants of carbapenem-resistant encoding genes, but only the major carbapenemase families.

## 5. Conclusions

The establishment of LFIA in routine microbiology labs could enhance the quality and speed of detection of the five principal carbapenemases in routine microbiology laboratories, delivering prompt and precise results at a reasonable expense. The LFIA demonstrated high diagnostic accuracy and strong agreement with PCR for the detection of key carbapenemase genes. With substantial to almost perfect kappa values and statistically significant concordance, the assay provides a reliable and rapid alternative to molecular methods.

Moving forward, however, further research is warranted to broaden the understanding of this assay’s utility. Specifically, future research is currently underway to perform a detailed, species-stratified analysis focusing on optimizing the LFIA’s performance within key bacterial classifications. Additionally, upcoming studies are planned to investigate the effect of different sample types on the LFIA’s diagnostic performance, which will be critical to fully determine its practical utility in diverse clinical settings.

## Figures and Tables

**Table 1:** Phenotypic detection of carbapenemase production by mCIM and eCIM tests among carbapenem-resistant isolates.

Organisms		mCIM		eCIM	
		Negative N = 13 (26%)	Positive N = 37 (74%)	Negative N = 29 (58%)	Positive N = 21 (42%)
*Klebsiella pneumonia* (n = 15*)*	n	4	11	9	6
	%	30.8%	29.7%	31%	28.6
*Acinetobacter baumannii* (n = 14)	n	4	10	7	7
	%	30.8%	27%	24.1%	33.3%
*Pseudomonas aeruginosa* (n = 11)	n	3	8	7	4
	%	23.1%	21.6%	24.1%	19%
*Escherichia coli* (n = 5)	n	1	4	4	1
	%	7.7%	10.8	13.8%	4.8%
*Enterobacter cloacae* (n = 3)	n	1	2	1	2
	%	7.7%	5.4%	3.4%	9.5%
*Klebsiella oxytoca* (n = 2)	n	0	2	1	1
	%	0%	5.4%	3.4%	4.8%

mCIM: modified carbapenem inactivation method; eCIM: EDTA-modified carbapenem inactivation method; n: number.

**Table 2:** Prevalence of carbapenemase-encoding genes detected by lateral flow immunoassay and PCR among carbapenem-resistant isolates.

	Total (n = 50)	*Klebsiella pneumonia* (n = 15)	*Acinetobacter baumannii* (n = 14)	*Klebsiella oxytoca* (n = 2)	*Escherichia coli* (n = 5)	*Pseudomonas aeruginosa* (n = 11)	*Enterobacter coloaca* (n = 3)	*p-Value*
	Carbapenemase genes by LFIA							
*bla_KPC_*	19 (38%)	9 (60%)	5 (35.7%)	1 (50%)	0 (0%)	3 (27.3%)	1 (33.3%)	0.203
*bla_NDM_*	27 (54%)	7 (46.7%)	10 (71.4%)	1 (50%)	2 (40%)	5 (45.5%)	2 (66.7%)	0.714
*bla_IMP_*	14 (28%)	4 (26.7%)	5 (35.7%)	1 (50%)	0 (0%)	3 (27.3%)	1 (33.3%)	0.696
*bla_VIM_*	24 (48%)	8 (53.3%)	6 (42.9%)	1 (50%)	3 (60%)	3 (27.3%)	3 (100%)	0.351
*bla_OXA-48_*	18 (44%)	8 (53.3%)	5 (35.7%)	1 (50%)	1 (20%)	1 (9.1%)	2 (66.7%)	0.139 *
	Carbapenemase genes by PCR							
*bla_KPC_*	17 (34%)	6 (40%)	5 (35.7%)	1 (50%)	1 (20%)	3 (27.3%)	1 (33.3%)	0.970
*bla_NDM_*	23 (46%)	7 (46.7%)	9 (64.3%)	1 (50%)	1 (20%)	3 (27.3%)	2 (66.7%)	0.380
*bla_IMP_*	12 (24%)	3 (20%)	5 (35.7%)	0 (0%)	0 (0%)	3 (27.3%)	1 (33.3%)	0.707
*bla_VIM_*	22 (44%)	7 (46.7%)	7 (50%)	1 (50%)	3 (60%)	1 (9.1%)	3 (100%)	0.042 *
*bla_OXA-48_*	15 (30%)	8 (53.3%)	3 (21.4%)	1 (50%)	1 (20%)	0 (0%)	1 (33.3%)	0.016 *

*p*: *p*-value for comparing between the studied groups; *: statistically significant at *p* ≤ 0.05.

**Table 3:** Performance of lateral flow assay in detection of single or multiple carbapenemase-encoding genes among carbapenem-resistant isolates.

Carbapenemase Genes by LFA (N = 50)	*Klebsiella pneumonia* (N = 15)	*Acinetobacter baumannii* (N = 14)	*Klebsiella oxytoca* (N = 2)	*Escherichia coli* (N = 5)	*Pseudomonas aeuroginosa* (N = 11)	*Enterobacter coloaca* (N = 3)
Negative N = 9 (18%)						
	1 (6.7%)	2 (14.3%)	1 (50%)	1 (20%)	4 (36.4%)	0 (0%)
Single gene N = 11 (22%)						
*bla_IMP_* _(N= 0)_	0 (0%)	0 (0%)	0 (0%)	0 (0%)	0 (0%)	0 (0%)
*bla_KPC_* _(N = 0)_	0 (0%)	0 (0%)	0 (0%)	0 (0%)	0 (0%)	0 (0%)
*bla_NDM_* _(N= 3)_	0 (0%)	1 (7.1%)	0 (0%)	1 (20%)	1 (9.1%)	0 (0%)
*bla_OXA-48_* _(N= 2)_	2 (13.3%)	0 (0%)	0 (0%)	0 (0%)	0 (0%)	0 (0%)
*bla_VIM_* _(N = 6)_	1 (6.7%)	2 (14.3%)	0 (0%)	2 (40%)	0 (0%)	1 (33.3%)
Double genes N = 12 (24%)						
*bla_IMP +_ bla_NDM_* _(N = 5)_	0 (0%)	3 (21.4%)	0 (0%)	0 (0%)	2 (18.2%)	0 (0%)
*bla_KPC +_ bla_NDM_* _(N = 3)_	2 (13.3%)	1 (7.1%)	0 (0%)	0 (0%)	0 (0%)	0 (0%)
*bla_KPC +_ bla_OXA-48_* _(N = 1)_	1 (6.7%)	0 (0%)	0 (0%)	0 (0%)	0 (0%)	0 (0%)
*bla_KPC +_ bla_VIM_* _(N = 1)_	0 (0%)	0 (0%)	0 (0%)	0 (0%)	1 (9.1%)	0 (0%)
*bla_NDM_ + bla_OXA-48_* _(N = 1)_	1 (6.7%)	0 (0%)	0 (0%)	0 (0%)	0 (0%)	0 (0%)
*bla_NDM_ + bla_VIM_* _(N = 1)_	0 (0%)	0 (0%)	0 (0%)	0 (0%)	1 (9.1%)	0 (0%)
Triple genes N = 8 (16%)						
*bla_IMP +_ bla_KPC +_ bla_OXA-48_* _(N= 1)_	0 (0%)	0 (0%)	0 (0%)	0 (0%)	1 (9.1%)	0 (0%)
*bla_KPC_ + bla_NDM +_ bla_OXA-48_* _(N= 1)_	0 (0%)	1 (7.1%)	0 (0%)	0 (0%)	0 (0%)	0 (0%)
*bla_KPC_ + bla_NDM +_ bla_VIM_* _(N= 2)_	1 (6.7%)	0 (0%)	0 (0%)	0 (0%)	1 (9.1%)	0 (0%)
*bla_KPC_ + bla_OXA-48 +_ bla_VIM_* _(N= 2)_	2 (13.3%)	0 (0%)	0 (0%)	0 (0%)	0 (0%)	0 (0%)
*bla_NDM +_ bla_OXA-48 +_ bla_VIM_* _(N= 2)_	0 (0%)	1 (7.1%)	0 (0%)	1 (20%)	0 (0%)	0 (0%)
Quadruple genes N = 7 (14%)						
*bla_IMP+_ bla_KPC +_bla_NDM +_ bla_VIM_* _(N= 2)_	2 (13.3%)	0 (0%)	0 (0%)	0 (0%)	0 (0%)	0 (0%)
*bla_IMP+_ bla_KPC +_bla_OXA-48 +_ bla_VIM_* _(N = 1)_	1 (6.7%)	0 (0%)	0 (0%)	0 (0%)	0 (0%)	0 (0%)
*bla_IMP+_ bla_NDM +_bla_OXA-48 +_ bla_VIM_* _(N = 2)_	1 (6.7%)	0 (0%)	0 (0%)	0 (0%)	0 (0%)	1 (33.3%)
*bla_KPC+_ bla_NDM +_bla_OXA-48 +_ bla_VIM_* _(N = 2)_	0 (0%)	1 (7.1%)	0 (0%)	0 (0%)	0 (0%)	1 (33.3%)
All five genes N = 3 (6%)						
*bla_IMP+_ bla_KPC +_bla_NDM+_ bla_OXA-48 +_ bla_VIM_* _(N = 3)_	0 (0%)	2 (14.3%)	1 (50%)	0 (0%)	0 (0%)	0 (0%)

**Table 4:** Performance of PCR in detection of single or multiple carbapenemase-encoding genes among carbapenem-resistant isolates.

Carbapenemase Genes by PCR N = 50	*Klebsiella pneumonia* (n = 15)	*Acinetobacter baumannii* (n = 14)	*Klebsiella oxytoca* (n = 2)	*Escherichia coli* (n = 5)	*Pseudomonas aeuroginosa* (n = 11)	*Enterobacter coloaca* (n = 3)
Negative N = 9 (18%)						
	1 (6.7%)	2 (14.3%)	1 (50%)	1 (20%)	4 (36.4%)	0 (0%)
Single gene N = 17 (34%)						
*bla*_IMP_ _(N = 2)_	0 (0%)	0 (0%)	0 (0%)	0 (0%)	2 (18.2%)	0 (0%)
*bla*_KPC (N= 3)_	0 (0%)	1 (7.1%)	0 (0%)	1 (20%)	1 (9.1%)	0 (0%)
*bla*_NDM (N= 2)_	0 (0%)	1 (7.1%)	0 (0%)	0 (0%)	1 (9.1%)	0 (0%)
*bla*_OXA-48 (N= 4)_	4 (53.3%)	0 (0%)	0 (0%)	0 (0%)	0 (0%)	0 (0%)
*bla*_VIM (N= 6)_	1 (6.7%)	2 (14.3%)	0 (0%)	2 (40%)	0 (0%)	1 (33.3%)
Double genes N = 9 (18%)						
*bla*_IMP+_ *bla*_KPC (N = 1)_	0 (0%)	0 (0%)	0 (0%)	0 (0%)	1 (9.1%)	0 (0%)
*bla*_IMP+_ *bla*_NDM (N = 2)_	0 (0%)	2 (14.3%)	0 (0%)	0 (0%)	0 (0%)	0 (0%)
*bla*_KPC+_ *bla*_NDM (N = 4)_	2 (13.3%)	1 (7.1%)	0 (0%)	0 (0%)	1 (9.1%)	0 (0%)
*bla*_NDM_+ *bla*_OXA-48 (N = 1)_	1 (6.7%)	0 (0%)	0 (0%)	0 (0%)	0 (0%)	0 (0%)
*bla*_NDM_+ *bla*_VIM (N = 1)_	0 (0%)	0 (0%)	0 (0%)	0 (0%)	1 (9.1%)	0 (0%)
Triple genes N = 8 (16%)						
*bla*_IMP+_ *bla*_NDM +_ *bla*_VIM (N = 2)_	1 (6.7%)	1 (7.1%)	0 (0%)	0 (0%)	0 (0%)	0 (0%)
*bla*_KPC_+ *bla*_NDM +_ *bla*_VIM (N = 1)_	0 (0%)	1 (7.1%)	0 (0%)	0 (0%)	0 (0%)	0 (0%)
*bla*_KPC_+ *bla*_OXA-48 +_ *bla*_VIM (N = 2)_	2 (13.3%)	0 (0%)	0 (0%)	0 (0%)	0 (0%)	0 (0%)
*bla*_NDM+_ *bla*_OXA-48 +_ *bla*_VIM (N = 3)_	1 (6.7%)	1 (7.1%)	0 (0%)	1 (20%)	0 (0%)	0 (0%)
Quadruple genes N = 5 (10%)						
*bla*_IMP+_*bla*_KPC +_*bla*_NDM +_ *bla*_VIM (N = 2)_	2 (13.3%)	0 (0%)	0 (0%)	0 (0%)	0 (0%)	0 (0%)
*bla*_IMP+_*bla*_NDM +_*bla*_OXA-48 +_ *bla*_VIM (N = 1)_	0 (0%)	0 (0%)	0 (0%)	0 (0%)	0 (0%)	1 (33.3%)
*bla*_KPC+_*bla*_NDM +_*bla*_OXA-48 +_ *bla*_VIM (N = 2)_	0 (0%)	0 (0%)	1 (50%)	0 (0%)	0 (0%)	1 (33.3%)
All five genes N = 2 (4%)						
*bla*_IMP+_*bla*_KPC +_*bla*_NDM+_ *bla*_OXA-48 +_ *bla*_VIM (N = 3)_	0 (0%)	2 (14.3%)	0 (0%)	0 (0%)	0 (0%)	0 (0%)

**Table 5:** Accuracy of lateral flow assay in comparison with PCR in detection of carbapenemase-encoding genes among tested isolates.

PCR		Lateral Flow Results		Kappa Value	*p-Value*	Sensitivity	Specificity	PPV	NPV	Accuracy
		Negative	Positive							
*bla*_IMP_	Negative (n = 38) Positive (n = 12)	35 (92.1%) 1 (8.3%)	3 (7.9%) 11 (91.7%)	0.793	<0.001	91.7%	92.1%	78.6%	97.2%	92%
*bla*_KPC_	Negative (n = 33) Positive (n = 17)	30 (90.9%) 1 (5.9%)	3 (9.1%) 16 (94.1%)	0.827	<0.001	94.1%	90.9%	84.2%	96.8%	92%
*bla*_NDM_	Negative (n = 27) Positive (n = 23)	23 (85.2%) 0 (0%)	4 (14.8%) 23 (100%)	0.841	<0.001	100%	85.2%	85.2%	100%	92%
*bla*_OXA-48_	Negative (n = 35) Positive (n = 15)	32 (91.4%) 0 (0%)	3 (8.6%) 15 (100%)	0.865	<0.001	100%	91.4%	83.3%	91.4%	94%
*bla*_VIM_	Negative (n = 28) Positive (n = 22)	25 (89.3%) 1 (4.5%)	3 (10.7%) 21 (95.5%)	0.839	<0.001	95.5%	89.3%	87.5%	96.2%	92%

**Table 6:** Accuracy of phenotypic methods in comparison with PCR in detection of carbapenem-resistant isolates.

PCR Genes	mCIM		Kappa Value	*p-Value*	Sensitivity	Specificity	PPV	NPV	Accuracy
	Negative N = 13	Positive N = 37							
Negative (n = 9) Positive (n = 41)	7 (53.8%) 6 (46.2%)	2 (5.4%) 35 (94.6%)	0.538	<0.001	94.6%	53.8%%	94.6%	53.8%	84%
	eCIM								
Negative (n = 9) Positive (n = 41)	9 (31%) 20 (69%)	0 (0%) 21 (100%)	0.274	0.006	100%	31%	100%	31%	60%

## Data Availability

The data presented in this study are available on request from the corresponding author due to [maintain privacy of data].

## Supplementary Materials

The following supporting information can be downloaded at: https://www.mdpi.com/article/10.3390/microorganisms14010031/s1, Table S1: Primers Used for the Detection of Carpabenemas Genes [12].
