# Supplementary material for: Comparison of Commercial Lateral Flow Immunochromatography with Phenotypic and Genotypic Assays for the Detection of Carbapenemase-Producing Gram-Negative Bacteria at Tanta University Hospitals
Source: Microorganisms. 2025 Dec 22;14(1):31. doi: 10.3390/microorganisms14010031 (PMC12843627; doi:10.3390/microorganisms14010031)
Supplement: Supplementary file 1 [file microorganisms-14-00031-s001.zip › microorganisms-4011759-supplementary.pdf]

Supplementary S1

Table S1. Primers Used for the Detection of Carpabenemas Genes. [12]

Target genes	Primer sequence	Amplicon size (pb)
bla$_{IMP}$	F: 5´- GGAATAGAGTGGCTTAAYTCTC-3´<br>R: 5´- GGTTTAAYAAAACAACCACC-3´	232
bla$_{KPC}$	F: 5´- CGTCTAGTTCTGCTGTCTTG-3´<br>R: 5´- CTTGTCATCCTTGTTAGGCG-3´	798
bla$_{NDM}$	F: 5´- GGTTTGGCGATCTGGTTTTC-3´<br>R: 5´- CGGAATGGCTCATCACGATC-3´	621
bla$_{OXA-48}$	F: 5´- GCGTGGTTAAGGATGAACAC-3´<br>R: 5´- CATCAAGTTCAACCCAACCG-3´	438
bla$_{VIM}$	F: 5´- GATGGTGTTTGGTCGCATA-3´<br>R: 5´- CGAATGCGCAGCACCAG-3´	390
